# Nutrition and Genetics in NAFLD: The Perfect Binomium

**DOI:** 10.3390/ijms21082986

**Published:** 2020-04-23

**Authors:** Marica Meroni, Miriam Longo, Alice Rustichelli, Paola Dongiovanni

**Affiliations:** 1 General Medicine and Metabolic Diseases, Fondazione IRCCS Ca’ Granda Ospedale Maggiore Policlinico, Pad. Granelli, via F Sforza 35, 20122 Milan, Italy; maricameroni11@gmail.com (M.M.); longo.miriam92@gmail.com (M.L.); rustichelli.alice@gmail.com (A.R.); 2 Department of Pathophysiology and Transplantation, Università degli Studi di Milano, 20122 Milano, Italy; 3 Department of Clinical Sciences and Community Health, Università degli Studi di Milano, 20122 Milano, Italy

**Keywords:** nonalcoholic fatty liver disease, nutrigenomics, nutrigenetics, nutriepigenomics, gene-diet interaction

## Abstract

Nonalcoholic fatty liver disease (NAFLD) represents a global healthcare burden since it is epidemiologically related to obesity, type 2 diabetes (T2D) and Metabolic Syndrome (MetS). It embraces a wide spectrum of hepatic injuries, which include simple steatosis, nonalcoholic steatohepatitis (NASH), fibrosis, cirrhosis and hepatocellular carcinoma (HCC). The susceptibility to develop NAFLD is highly variable and it is influenced by several cues including environmental (i.e., dietary habits and physical activity) and inherited (i.e., genetic/epigenetic) risk factors. Nonetheless, even intestinal microbiota and its by-products play a crucial role in NAFLD pathophysiology. The interaction of dietary exposure with the genome is referred to as *‘nutritional genomics,’* which encompasses both *‘nutrigenetics’* and *‘nutriepigenomics.’* It is focused on revealing the biological mechanisms that entail both the acute and persistent genome-nutrient interactions that influence health and it may represent a promising field of study to improve both clinical and health nutrition practices. Thus, the premise of this review is to discuss the relevance of personalized nutritional advices as a novel therapeutic approach in NAFLD tailored management.

## 1. Introduction

Nonalcoholic fatty liver disease (NAFLD) is now considered the most frequent cause of liver disorders worldwide, affecting more than one third of the general population [1]. Since it has been reaching global epidemic proportions, it represents one of the major social, economic and public health issues in western countries [1]. NAFLD is defined by hepatic fat accumulation that exceeds 5% of liver weight, in absence of alcohol abuse, and it entails a broad spectrum of conditions, spanning from simple and uncomplicated steatosis to nonalcoholic steatohepatitis (NASH), which is characterized by hepatocyte ballooning, lobular inflammation and fibrosis that could worsen into cirrhosis and hepatocellular carcinoma (HCC) [2,3].

NAFLD is epidemiologically associated with obesity, type 2 diabetes (T2D), and metabolic syndrome (MetS) features [4] and its pathogenesis is closely entangled with increased adiposity, insulin resistance (IR) and dyslipidemia [5]. Indeed, dietary habits such as excessive caloric intake, high fructose consumption and poor physical activity represent paramount risk factors for this condition [6]. 

Furthermore, the inter-individual susceptibility to develop NAFLD may be partially explained by inherited factors [7]. Single nucleotide polymorphisms (SNPs) in genes regulating hepatic lipid handling, including *Patatin-like Phospholipase Domain-containing 3* (*PNPLA3*), *Transmembrane 6 Superfamily Member 2* (*TM6SF2*) and *Membrane Bound O-acyltransferase Domain-containing 7* (*MBOAT7*), have been associated with NAFLD predisposition and progression towards NASH and fibrosis [7]. However, less than 10% of inherited variability is explained by these common variants. In particular, many of the phenotypic discrepancies may also result from complex gene-environment interactions, explained by epigenetic mechanisms, hereditable but reversible modifications that modulate the transcriptome in response to environmental cues, without altering DNA sequence [8]. Epigenetics encompasses a wide number of events such as alterations of DNA nucleotides (i.e., methylation of CpG dinucleotides, known as CpG islands), modifications of histones and regulation of transcription by altering mRNA stability through small RNA molecules such as microRNAs (miRNAs). Therefore, it may provide a new perspective in NAFLD pathogenesis, diagnosis and management [9]. Nonetheless, among the plethora of multiple parallel hits, recent evidence has pointed out the role of intestinal dysbiosis, microbial metabolites and enhanced intestinal permeability in the pathophysiology of fatty liver [10,11].

Currently, no therapeutic consensus exists for the treatment of NAFLD patients, and lifestyle intervention with a focus on healthy eating and regular physical exercise and weight loss remain the mainstay in the management of patients with NAFLD. Indeed, effective and sustained weight loss has been associated with marked improvement in glycemic control, hepatic insulin sensitivity, liver enzymes and liver histology [6]. However, it has been demonstrate that each individual differs in response to diet, according to the genetic background [6]. The interaction between nutritional environment and inherited factors is being referred to as *‘nutritional genomics’* or *‘nutrigenomics,’* which encompasses both *‘nutrigenetics’* and *‘nutriepigenomics’* [12,13]. Nutritional genomics may represent a promising field of study to improve both clinical and health nutrition practices, establishing genome-based dietary guidelines for disease prevention, individualized nutrition therapy for disease management and targeted public health nutrition interventions [13]. It is focused on revealing the biological mechanisms that entail both the acute and persistent genome-nutrient interactions that influence health [13]. 

For this reason, this review aimed to address to the relevance of personalized nutritional approaches in the tailored NAFLD clinical management and its pivotal role as therapeutic strategy to ameliorate liver damage and to avoid its progression towards end-stage conditions. Moreover, the discovery of possible nutrigenomic approaches may contribute to explain how dietary habits differently impact on health depending on the individual’s genetic makeup. 

## 2. Methodological Approaches to Nutritional Genomics

In the last decades, the prevalence of metabolic disorders (e.g., NAFLD, obesity and T2D) has exponentially increased in Western countries. This escalation is strictly correlated with changes in dietary habits. Indeed, the Western diet is evolutionally modified, replacing fruits, vegetables, proteins and omega-3 fatty acids with saturated and trans-fat, omega-6 fatty acids, carbohydrates and high-energy nutrients [14]. It has been demonstrated that nutritional and lifestyle interventions exert beneficial effects on NAFLD outcomes and its comorbidities. Nutritional genomics studies the impact of nutrients on gene expression, genome evolution and selection, genome mutation rate, and genome reprogramming [13]. It entails even the detrimental effect exerted by specific macro and micronutrients on DNA metabolism, addressing mainly their role in DNA synthesis, degradation, repair and alteration. In turn, the genomic evolution and selection may contribute to the genetic variations observed within genetically different ethnicities. The deep-knowledge of diet-genome interactions will allow to apply new approaches on prevention and treatment of chronic disorders by using precision nutrition, which might be included in the personalized medicine therapy. However, the amount of studies is scarce and nutrigenomic research remains largely inconclusive. Therefore, there is an urgent need to increase the number of experimental data in order to unravel these mechanisms and to discover novel appealing candidate biomarkers for diagnosis as well as to introduce nutraceutical products as preventive or therapeutic strategy [13]. This paragraph aims to describe the current strategies in this scenario, and to elucidate appropriate methodological approaches to nutrigenomics, thus providing the finest interpretation of the nutritional effect on health outcomes and nutritional guidelines. Three main study approaches of nutrigenomics will be covered: (1) the effects of nutrients on DNA metabolism; (2) the study of genetic variability response towards nutrition named nutrigenetics; (3) the effect of nutrients on genetic expression. 

An important aspect of nutrigenomics is the effectiveness of nutrients (especially micronutrients) on DNA metabolism, even though it is not deeply investigated. Some evidence supports the notion that several micronutrients are required to maintain DNA homeostasis, as they are cofactors of a variety of enzymes involved in DNA synthesis and repair [15]. Thus, nutritional deficiency of these essential micronutrients could induce a strong DNA modification comparable to that observed after DNA exposure to mutagenic substances or radiations [16]. Fenech et al. tested the association between dietary micronutrient intake and genome stability in a human trial, using cytokinesis-block micronucleus assay (CBMN) in lymphocytes. This test is based on the concept that the formation of micronuclei during cytokinesis, instead of two whole nuclei, is an indicator of DNA damage. These authors demonstrated that a higher intake of several micronutrients (i.e., calcium, folate, nicotinic acid, vitamin E, retinol, β-carotene) is associated with a decrease in micronuclei formation, and therefore, reduced genome damage [17]. This is a highly innovative and reliable method to study genome alterations in human samples in a simple and non-invasive fashion. The study of micronuclei formation as a signature of genomic derangement has significant implications for tumorigenesis and cancer research [16]. Another method to investigate genomic stress is the assessment of telomere length. Indeed, telomeres shortening is a hallmark of aging and of several chronic diseases, among which NAFLD [18]. A broad number of studies demonstrated that dietary habits have either protective or noxious effects on telomere length. For instance, high consumption of vegetables and omega-3 fatty acids were associated with longer telomeres, whereas higher intake of saturated fatty acids was correlated with shorter ones [19]. Therefore, since nutritional pattern is highly relevant in NAFLD pathogenesis, it can be hypothesized that telomere shortening, and hence genome stress, in NAFLD patients may be affected by dietary habits. 

Another area of interest of nutrigenomics is represented by nutrigenetics. The latter entails the study of the effect of a genotype (e.g., the presence of SNPs or other genetic variations) towards specific dietary patterns. Indeed, each subject could respond differently to nutritive substances, and genetic variations within different human populations are a consequence of the adaptive evolution to specific dietary habits. Common SNPs in DNA sequence constitute the primary example of genetic variation. They arise from a process of DNA mutation and subsequent selection in the populations. Nutritional environment intervenes in this evolutionary process, precipitating the expansion of DNA mutations within the subjects. For instance, Vitamin B deficiency as well as high iron intake strongly impair DNA synthesis and stability, enhancing the rate of germ line and somatic mutations. Likewise, derangements in folate metabolism enhance the risk of several diseases such as cancers, cardiovascular disorders and neurological disorders [20]. Genetic variants that alter folate-dependent enzymes can confer an increased or decreased susceptibility to develop these abnormalities. Indeed, the common SNP in the *Methylenetetrahydrofolate Reductase* (*MTHFR*) gene, 677C>T enhances the risk for neural tube anomalies [21] and metabolic disorders such as NAFLD [22]. However, the same polymorphism has been also demonstrated to be protective against colon cancer in folate-supplemented individuals [23]. The prevalence of this SNP is highly heterogeneous among the different ethnicities (the allele frequency is 20% in Hispanics, rare in Africans) as a consequence of the selection due to the nutritional environment [24]. However, several genetic variants affecting nutrient utilization that are penetrated in the population through positive selection are to date considered inherited risk factors for metabolic disorders (i.e., C282Y variant in *HFE* gene) [25]. 

Due to the complexity of gene-diet interactions, recent steps forward have been carried out to promote nutritional research. The study of the effect of genetic variations on dietary intake in the field of metabolic disorders is possible through genetically edited in vitro models such as cell lines, co-cultures and three-dimensional (3D) cultures and the use of mice models (e.g., knock-out, knock-in) bearing the mutations of interest. In these models, the harmful effects of fatty acids overload or obesogenic/steatogenic diets, respectively, could be influenced by the genetic background and potentially be reverted through healthy micro/macronutrients supplementation [26]. In particular, the breakthrough in the field of genome editing reached the top with the introduction of Clustered regularly interspaced short palindromic repeats (CRISPR)/Cas9 for both in vivo and in vitro models, which offers the advantage to reduce the off-target effects occurring with other techniques. Furthermore, human-derived organoids from stem cells represents innovative attractive models, capable to differentiate into multiple cell lineages according to the culture conditions [26]. 

Nutrigenomic study investigates also the effect of nutrients on gene expression. The primary goals of this nutrigenomic approach encompass the following steps: (1) to identify the transcription factors that are sensitive to a specific nutrient and their target genes; (2) to find out which signaling pathways are activated as a consequence of the targeted genetic transcription, and therefore, possible metabolites and pathophysiological processes involved; (3) to define cell- and organ-specific response to nutrients and the relative consequences [27]. These aims are pursued with the implementation of ‘omics’ technologies, among which genomics, transcriptomics, proteomics, metabolomics and system biology. The latter integrates bioinformatics to “omics” approaches, allowing to identify the functional roles of different genes in a dynamic network interaction, thus giving rise to the new functional genomics era [28,29]. Notwithstanding, the application of high-throughput “omics” methods could run into several limitations: (1) gene expression is often cell/tissue specific, and therefore, a limitation could be represented by harvesting samples through invasive methods, especially in human studies; (2) analysis and interpretation of data, which sometimes requires advanced bioinformatic competences [29]. 

In the past years, DNA microarray technique was the most exploited tool for the analysis of transcriptomic data and a promising method for nutrigenomic studies, as it may assess the effects of macro and micronutrients on the entire transcriptome [28]. This information will allow to translate in detail whether nutrients’ effects on the transcriptome mirror the consequences on proteome and, in turn, what downstream pathways are activated. A more innovative transcriptomic tool is RNA sequencing (RNAseq), which is emerging as an alternative to DNA microarray. Indeed, RNAseq experiments are more informative, as they can cover a larger spectrum of RNAs compared to microarrays, also detecting very low expressed RNAs [30]. 

Once it is clear which transcripts are regulated by nutrients, the function of these transcripts needs to be investigated; namely, which proteins they codify for. Proteomics is the analysis of protein expression profile, which reflects cellular activity. Proteomics studies exploit a wide variety of antibodies-based techniques, such as electrophoretic separations and blotting or enzyme-linked immunosorbent assay (ELISA). However, a more innovative and advance method for proteome analysis is mass spectrometry (MS), which simultaneously detects all the proteins synthetized [31]. MS is based on the ionization of molecules (e.g., peptides, proteins) that are detected according to their mass to charge ratio (m/z). In the past years, MS has played a major role in the development of proteomics because of its sensitivity and specificity for protein identification and characterization. Moreover, another important advantage of MS is that it allows to analyze all sorts of samples in liquid and solid form, such as tissue samples. In this case, MS not only can give quantitative but also spatial information about protein expression [32]. Additionally, MS is a useful state-of-the-art tool to study the metabolome. Metabolomics, the most novel “omics” approach used in nutrition, studies the whole metabolism, from the single cell to the entire organism, and it represents the ending result of the modifications in gene expression. Metabolomics can be trickier than proteomics because metabolites have different chemical structures, and therefore, no method makes possible to characterize the whole metabolome at once. Moreover, the ending molecular result of metabolism can be extremely sensitive to several environmental factors, not only nutrients, which can be misleading. Another important method used in metabolomics is Nuclear Magnetic Resonance (NMR) spectroscopy, which enables not only the quantification of metabolites, but also their chemical structure. However, sometimes, NMR can reliably detect metabolites in high concentrations. Both MS and NMR allow the assessment of metabolites from biological fluids and tissue samples [33].

An integrative methodological approach was used by Xie et al. when they explored hepatic transcriptome and metabolome alterations in male Wistar rats fed high fat diet (HFD) for 16 weeks. A transcriptome analysis demonstrated that 130 genes were differentially expressed in the livers resected from HFD-fed rodents, compared to those fed standard chow. The majority of differentially expressed genes were intertwined in lipid synthesis and metabolism and in energy utilization. Moreover, these authors investigated the lipid composition in both serum and hepatic tissues through gas-chromatography/mass spectrometry as metabolomics approach, revealing an unbalanced composition of lipids and an increase in saturated and monounsaturated fatty acids. These comparative observations revealed that hepatic fatty acid utilization through β-oxidation is inhibited and *de novo* lipogenesis (DNL) is enhanced after HFD administration in rodents [34].

Nonetheless, an unbalanced diet may induce inflammatory and metabolic stress as well, as a result of the impaired interactions between transcription factors and nuclear receptors, thus affecting the transcription and translation cascades. Therefore, not only should the direct effect of nutrients on transcription be considered, but also the indirect effect caused by inflammatory and metabolic stress. A good example to explain this effect is the regulation of Peroxisome proliferator-activated receptor-α (PPARα). In the liver, PPARα is a transcription factor that promotes fatty acid intake from the bloodstream and their catabolism, through β-oxidation processes. Obesity and T2D are characterized by a chronic status of low-grade inflammation, implicating the release of cytokines, which in turn lead to PPARα downregulation (inflammatory stress). Moreover, in obese and diabetic individuals, the level of fatty acids is constantly elevated, constituting a metabolic stressor, further compromising PPARα activity [27]. Thus, another important goal of nutrigenomic research is to elucidate the double interaction of metabolic and inflammatory stressors triggered by unhealthy diet, with physiological pathways. 

The deep understanding of the genome-diet interactions may lead to the identification of non-invasive biomarkers representative of the human nutritional status, possibly revealing individual dietary habits in a more precise and objective way compared to questionnaires. Moreover, nutritional biomarkers could be indicators of pathophysiological status. Well-established examples are the plasma levels of fasting glucose, associated with diabetes, or the plasma cholesterol and triglycerides, linked with cardiovascular disorders [30]. In the case of NAFLD, a potential nutritional biomarker could be the serum levels of saturated fatty acids. Together with transcriptomic, proteomic, metabolomic and lipidomic biomarkers, epigenetic modifications can be implemented as nutritional biomarkers as well [30]. Indeed, DNA methylation, miRNAs or long-chain non-coding RNAs, modulated by dietary patterns, have emerged as mRNA transcription regulators of pivotal importance in pathophysiological processes [30], as precisely summarized in the following sections. 

Therefore, considering the genetic background of an individual, its epigenetic signature and its phenotypic hallmarks, through the interaction of all ‘omics,’ methodological approaches may define an integrated Personal Omic Profile (iPOP), acquiring an enormous potential of omics integration in medical researches, in monitoring health and personalized interventions [35].

## 3. Nutrigenetics: New Field to Customize Intervention Strategies

Although obesity and IR are the most prevalent risk factors for NAFLD pathogenesis, hepatic fat content varies substantially among individuals with equivalent adiposity, indicating that other risk factors may contribute to this condition. Indeed, epidemiological, familial and twin studies provide evidence of a strong heritability of hepatic lipid accumulation [36,37]. In 2009, Schwimmer et al. demonstrated that parents and siblings of overweight children with NAFLD have an increased predisposition to develop a fatty liver compared to obese children without NAFLD [38]. Nonetheless, it has been demonstrated that familial NASH clustering is frequent, reaching 18% of patients having a similarly affected first degree relative [39]. 

Furthermore, there is a huge inter-ethnic variability in the predisposition towards NAFLD [36,37]. Two large multi-ethnic population studies conducted in the United States revealed an higher risk of NAFLD onset in Hispanic individuals than those of European descent, whereas African-Americans are protected irrespectively of diabetes, excess in body weight and socioeconomic factors, consistently, with a key role of heritability [7]. In particular, African-Americans differed in the metabolic response to obesity and IR when compared to either Hispanics or Caucasians, appearing more resistant to triglyceride (TG) accumulation both in the adipose tissue and in the liver [40]. 

To date, several inherited risk factors have been recognized to be implicated in genetic susceptibility to develop NAFLD and its progressive forms [7]. However, the major common predictors of the inherited predisposition to severe NAFLD are the variants in *PNPLA3, TM6SF2*, *Glucokinase Regulator* (*GCKR*) and *MBOAT7* genes.

Nutritional genetics or nutrigenetics highlights the impact of human genetic variations on nutrient utilization/metabolism/processing, food tolerances and nutrient requirement [13]. It tells us how an individual’s genetic background will shape the risks and benefits of consuming different types of foods and nutrients [14]. Scientific advances in this field might pave the way in the future to NAFLD prevention and treatment, predicting the individual risk, explaining personal pathophysiology and customizing nutritional management. Furthermore, to understand the biological mechanisms entailing genome-nutrient interactions may constitute a revolutionary frontier in the knowledge of how common chronic human diseases are initiated or accelerated by nutrient exposure. Examples of possible gene-diet interactions and personalized therapeutic interventions to prevent NAFLD onset and progression are summarized in the paragraphs below. 

### 3.1. PNPLA3: An Appealing Genetic Sensor of Dietary Compounds

The main genetic determinant of the inter-individual and ethnicity-related differences in hepatic fat content is the rs738409 C>G variant in *PNPLA3* (also known as adiponutrin), encoding the amino acid substitution Isoleucine to Methionine at the position 148 (referred to as p.I148M) [41]. Carriers of the G at-risk alleles are more prevalent in Hispanics than in Europeans and less frequent in African-Americans, possibly explaining the inter-ethnic susceptibility to NAFLD. PNPLA3 is an intracellular membrane lipase, localized mainly in the endothelial reticulum (ER) and at the surface of the lipid droplets in hepatocytes, adipocytes and in hepatic stellate cells (HSCs) [42,43]. This inherited variant strongly impacts on the entire spectrum of liver damage related to fatty liver, encompassing NASH, severe fibrosis and HCC, even influencing the response to therapeutic approaches [44]. The underlying mechanisms whereby the p.I148M variant induces steatosis development seems to be related to the accumulation of the PNPLA3 mutated protein on the lipid droplet surface. This event may be due to the lesser accessibility to ubiquitin ligases and to impaired proteasomal degradation of the I148M protein forms [45]. Thus, it interferes with lipid remodeling in fatty-laden hepatocytes, even inhibiting the activity of other lipases (i.e., PNPLA2), and accordingly, reducing TG turnover and dismissal [46]. Indeed, chronic overexpression of the PNPLA3 I148M variant in mice impairs TG hydrolysis and depletes long-chain PUFA [47]. In HSCs, conversely, the PNPLA3 I148M variant alters retinol secretion, potentially contributing directly to fibrogenesis and carcinogenesis, increasing the risk for cirrhosis and HCC development, independently of the predisposition to steatosis [48,49,50]. The size effect of the p.I148M variant on the risk of NAFLD is the strongest ever reported for a common variant, modifying the genetic susceptibility of NAFLD and liver disease progression [6]. 

It has been demonstrated that the accumulation of the mutated I148M protein may be triggered by environmental factors. At a nutritional level, PNPLA3 expression is transcriptionally modulated by the activation of the Sterol regulatory element-binding protein 1 (SREBP1c)/Liver X Receptor (LXR) pathway induced by hyperinsulinemia and by carbohydrate feeding. Moreover, PNPLA3 protein degradation can be inhibited by the presence of fatty acids [51,52]. 

In an intriguing study, Santoro et al. revealed the presence of an interaction between *PNPLA3* rs738409 variant and the dietary ratio of omega-6/omega-3 polyunsaturated fatty acids (PUFA) on hepatic fat content and alanine aminotransferase (ALT), in 127 pediatric NAFLD patients of various ethnicities (58 Caucasians, 30 African-Americans and 39 Hispanics) [53]. Patients homozygous for the minor G allele ameliorated steatosis and liver enzymes after omega-3 consumption, due to the inhibition of hydrolyzing n-9 fatty acids in presence of the p.I148M variant. Indeed, the presence of the rs738409 variant may hinder the PNPLA3 hydrolytic function, lowering the protein ability in hydrolyzing the n-9 of about 15% [54]. The n-9 can be derived directly from diet (i.e., meat, olive oil, sesame oil, almonds and avocados) or they are synthesized starting from n-6 PUFA [55]. All these observations suggest that increasing dietary intake of foods rich in n-3 PUFA such as salmon, tuna and flaxseed oil, or supplementing the diet with n-3 PUFA respect to n-6 PUFA could provide a targeted therapy to subjects with NAFLD homozygous for the G allele [53]. Indeed, n-6 overload may serve as substrate for TG synthesis, further accelerating hepatic steatosis and in the meantime, it delays the hydrolytic function of the PNPLA3. The remnant n-6 not incorporated into TG may lead to the over-production of pro-inflammatory omega 6-derived species, triggering the switching from simple steatosis to NASH. Moreover, an excess of free fatty acids (FFAs) enhanced hepatic TG accumulation in the presence of the p.I148M variant, significantly down-modulating lipid hydrolyzation [56]. 

Conversely, in the double-blind placebo controlled WELCOME trial (NCT00760513), 103 adult patients with NAFLD were randomized to receive a supplementation of omega-3 fatty acids (long chain PUFA), including docosahexaenoic (DHA) and eicosapentanoic acid (EPA) or placebo for 15–18 months. *PNPLA3* homozygous patients displayed an independent association with a decrease percentage of DHA tissue enrichment during the trial but did not with changes in serum TG concentration [57]. Consistently, in another randomized controlled trial (NCT00885313), it was tested whether the PNPLA3 I148M variant is associated with the response to DHA (250 or 500 mg/day) in 60 children with NAFLD for 24 months. This study demonstrated that the 148M allele is associated with no beneficial effect of DHA supplementation regarding liver fat, showing a double risk of severe steatosis at the end of the trial [58]. These findings clarify the data reported by Santoro et al., showing that PNPLA3 is implicated in omega-3 fatty acid mobilization in the livers [53]. In turn, omega-3 fatty acids may down-modulate SREBP1c expression and PNPLA3 148M variant is associated with lower DNL despite the substantial increased hepatic fat content, thus explaining the lower response to DHA+EPA therapy in these patients [59].

Furthermore, in 153 overweight Hispanic children, Davis et al. revealed that the hepatic fat fraction in carriers of the GG genotype is strongly influenced by carbohydrate and total sugar dietary intake (clinical trials NCT00697580 195-1642394A1 and NCT00693511) [60]. Indeed, high dietary sugar consumption may induce SREBP1c and, in turn, PNPLA3 mutated protein expression exacerbating fat deposition [60]. Moreover, low carbohydrate diet consumption for 6 days exerts a greater impact on liver fat content in PNPLA3 148MM subjects (trial registered as 233,775 at www.hus.fi) [61]. Similarly, in 200 adolescents, Nobili et al. demonstrated that the moderate intake of sweetened beverages led to increased hepatic fat deposition in *PNPLA3* homozygous, and hampered consumption of vegetables declined the risk of severe steatosis, thus reporting a possible interaction between PNPLA3 I148M and dietary components regarding the severity of steatosis [62]. 

In obese children carrying wild-type allele, weight loss predisposes to more effective benefits on intra-hepatic fat content and liver enzymes [63].

Overall, this evidence may support the notion that dietary interventions may exert various effect in NAFLD patients depending on their genetic background. 

### 3.2. TM6SF2: Two Sides of the Same Coin 

The low-frequency rs58542926 C>T variant in *TM6SF2*, encoding the loss-of-function mutation referred to as p.E167K is another determinant of progressive NAFLD [64]. The likely mechanism through which the E167K variant predisposes to an increase in hepatic fat content seems to be related to the retention of lipids and the impairment of very low-density lipoprotein (VLDL) release by the liver. Moreover, *TM6SF2* rs58542926 variation modulates the hepatic fat content, impairing lipid synthesis from PUFAs [65]. Indeed, TM6SF2 is mainly implicated in TG-rich lipoprotein lipidation and secretion and it is mainly expressed in small intestine and in the liver. Patients carrying the T minor allele display increased aminotransferase concentrations, low serum lipoproteins and steatosis development. Conversely, by reducing circulating lipids, the E167K variant decreases the risk of cardiovascular events in both children and adults [66,67,68]. 

O’Hare et al. demonstrated that subjects carrying the T allele display improved fasting and postprandial lipid profiles, even after a high fat challenge, in a cohort of 3556 individuals enrolled in the Amish Complex Disease Research Program (ACDRP). In addition, these authors investigated the impact of TM6SF2 deficiency in a zebrafish model and in cultured human Caco-2 cell lines, in which they revealed that *TM6SF2* loss of function decreased lipid clearance and, in turn, favors TG accumulation and ER stress in enterocytes. All these findings support the crucial role of TM6SF2 in dietary lipid metabolism in small intestine, exerting a similar function in the lipidation and release of both hepatically- and intestinally-derived TG-rich lipoproteins [69]. Nonetheless, concerning the postprandial glucose homeostasis, Musso et al. correlated the presence of *TM6SF2* variation with increased hepatic and adipose IR, impaired pancreatic β-cell function and higher muscle insulin sensitivity and whole-body fat oxidation rate [70]. Moreover, the T-allele entailed a lower postprandial lipemia, a less atherogenic lipoprotein profile, and a postprandial cholesterol redistribution from smaller atherogenic lipoprotein subfractions to larger intestinal and hepatic VLDL subfractions [70]. Therefore, these authors demonstrated that the *TM6SF2* C>T polymorphism affects not only the postprandial lipoprotein metabolism, but also the nutrient oxidation, glucose tolerance and the gastrointestinal response to fat ingestion [70]. 

However, Krawczyk et al. reported that *TM6SF2* variant as well as *PNPLA3* variation did not impair the response to low-calorie four-month dietetic intervention in 143 NAFLD patients [71]. Indeed, the dietary interventions improved phenotypic traits, steatosis and ALT concentrations independently of the presence of these two variants [71]. Finally, Dongiovanni et al. revealed that the rs58542926 *TM6SF2* variant did not impact on the beneficial effect of statins administration in NAFLD patients [72], opening the possibility to treat T allele carriers with statins to ameliorate liver damage, even if the lipid profile of these patients does not encourage this therapeutic strategy [67].

### 3.3. GCKR: A Potential Carbohydrates’ Modulator to Improve Liver Damage 

Besides *PNPLA3* and *TM6SF2* variations, the common loss-of-function rs1260326 variant in the *GCKR* gene, encoding the p.P446L substitution, has been widely associated with increased fasting TG concentrations, steatosis and liver damage. This protein physiologically regulates glucose influx into the hepatocytes, mediating the activation of DNL. The alteration of GCKR impairs its ability to negatively modulate glucokinase, constitutively activating the hepatic glucose uptake. On one side, this event may improve circulating fasting glucose and insulin concentrations; on the other, it may favor glycolysis and steatosis onset, by providing malonyl-CoA as substrate for DNL and by blocking fatty acid oxidation [73,74,75]. 

Several studies have explored the interaction between *GCKR* rs1260326 variant and the environmental factors [74,76,77]. In an open-labeled and single-arm clinical trial, Kaliora et al. demonstrated that T allele carriers respond better to nutritional intervention in terms of fasting blood glucose levels compared to non-carriers, in 44 overweight adults with NAFLD which have been received dietary counseling for six months [76]. For instance, whole-grain supplementation is associated with lower fasting glucose and insulin concentrations independently of demographics, other dietary and lifestyle factors and BMI [78]. An interesting interaction between the GCKR variation and whole grain intake has been proposed by Nettleton et al. These authors demonstrated that a greater whole-grain intake is even more strictly associated with the reduction in fasting insulin concentrations in patients carrying the at risk allele [78]. 

Moreover, Santoro et al. reported for the first time the rate of DNL through incorporation of deuterium into the palmitate contained in the VLDL after the administration of a carbohydrate drink (75 g glucose and 25 g fructose) in obese adolescents. These authors demonstrated that the *GCKR* rs1260326 variation in homozygosity increased hepatic lipid synthesis in obese adolescents, as a result of the enhanced glycolytic carbon flux to TG formation [74]. This study was the first that revealed in pediatric subjects that a common variant might favor steatosis onset by enhancing the ability of the liver to convert carbohydrate into TG, raising the possibility to decrease glucose and fructose dietary intake in patients homozygous for the rs1260326 variant to improve liver damage [74]. Indeed, it has been elucidated that the adherence to the Mediterranean diet (MedDiet) may modulate the detrimental effect of rs1260326 variant by hampering circulating lipids [79]. Consistently, the association between *GCKR* genotype and serum total cholesterol in children is modulated by dietary monounsaturated fatty acid relative to saturated fatty acid (MUFA:SFA) ratio [80].

### 3.4. MBOAT7 as a Novel High-Sensitive Converter of Nutritional Substrates

In 2005, a genome-wide association study (GWAS) revealed that the novel common rs641738 C>T variant in the *MBOAT7-TMC4* locus on chromosome 19 increased the susceptibility to alcoholic cirrhosis in heavy drinkers [81]. Then, it has been demonstrated that rs641738 variation is associated with a strong predisposition towards hepatic fat accumulation and to the entire spectrum of liver damage related to NAFLD, including HCC [82,83]. Notably, it has been identified as a risk factor for fibrosis development in viral hepatitis B and C, possibly representing a common modifier of liver damage [84,85]. 

MBOAT7, also known as lyso-phosphatidylinositol (lyso-PI) acyl-transferase1 (LPIAT1), encodes for an enzyme member of the “Lands’ Cycle” of phospholipid acyl-chain remodeling of the membranes. It is mainly localized in the mitochondria-associated membrane (MAM), which is the membrane bridging ER and mitochondria in which the fat biosynthesis and lipid droplets formation occurs. It conjugates an acyl-CoA to the second acyl-chain of lyso-phospholipids, using as preferential substrate the arachidonoyl-CoA. Thus, it regulates the desaturation of phospholipids and the amount of free arachidonic acid, a precursor of proinflammatory mediators (eicosanoids) [86]. It has been made clear that the likely mechanisms behind the association between the rs641738 variant and liver damage is related to the hampered hepatic gene and protein expression of MBOAT7, determining changes in phosphatidylinositol species [82,87]. Furthermore, we have recently pointed out that hepatic MBOAT7 down-regulation is a dysfunctional response to inherited or diet-induced hyperinsulinemia and that its reduced expression is enable to induce intracellular fat accumulation in clinical samples, in in vivo models of NAFLD and in genetically edited HepG2 cells (*MBOAT7^-/-^*) [87]. Specifically, in the overweight adults, MBOAT7 is hampered in the presence of hyperinsulinemia and severe liver damage, independently of the genetic background. These findings have been even more clear in experimental models of NAFLD, in which the reduction of MBOAT7 expression is stronger during obesity and IR. Consistently, even fructose administration reduces hepatic Mboat7 in rodents (unpublished data). 

During post-prandial or pathological hyperinsulinemia and in carriers of the T allele, MBOAT7 expression and function is impaired, contributing to the increase of saturated phospholipids. The accumulation of these compounds, mainly phosphatidylinositol species, may be shunted to the synthesis of saturated and mono-unsaturated TG, further corroborating fat deposition. This process requires the upregulation of the fatty acid transporter (FATP1) and the consequent fatty acid uptake. According to this notion, *FATP1* genetic deletion rescued the intracellular fat accumulation and the increased lipogenesis observed in the *MBOAT7^-/-^* background [87]. Our novel evidence may introduce the idea that a well-balanced diet, characterized by low carbohydrate and saturated fatty acid consumption, may influence the genetic susceptibility to develop fatty liver in carriers of the rs641738 variant. These notions pave the way to improve our knowledge about the influence of macro and micronutrients on this genetic substrate, emphasizing the concept that it may provide a new example of gene-environment interaction, which should be deeply explored in future studies.

### 3.5. Other NAFLD Genetic Risk Factors Responsive to Diet

In 2014, Miele et al. conducted for the first time a case–control study on the effect of metabolic gene polymorphisms, nutrition and their interaction on the risk of NAFLD in 294 Italian cases and 359 controls [88]. These authors reported that young adults and males display an increased risk of NAFLD, as well as those subjects which consume an unbalanced diet. Oxidative stress and mitochondrial dysfunction are hallmarks of severe NAFLD. Therefore, the authors examined the interaction between dietary factors and inherited variants in genes that exert key roles as antioxidant defense, such as *glutathione S-transferase Mu 1* (*GSTM1*), *glutathione S-transferase theta 1* (*GSTT1*), cytochrome P450 superfamily members and *sulfotransferase 1A1* (*SULT1A1*). They found that these inherited factors significantly interact with high fruit intake (more than two fruits/day) or high grilled meat/fish consumption (more than once per week), exasperating the risk of developing NAFLD and suggesting a possible role of aromatic hydrocarbons in liver steatosis [88].

Another important study investigated the response to diets depending on individual genetic backgrounds by exploiting the nutrient-induced insulin output ratio (NIOR) in order to identify patients phenotypically sensitive to glucose or fat in the diet [89]. In this paper, the authors examined the polymorphisms linked with NIOR according to their impact on the output of insulin after a meal in 171 patients, after which they create a nutritional plan to follow for 6 months. The reduction of total and hepatic fat mass was most effective in the group in which the amount of dietary fat or sugar was adjusted to gene polymorphisms, among which *Adrenergic receptor* (*b3AR*), *Tumor necrosis factor α* (*TNFα*), *Apolipoprotein C3* (*Apo C3*), *Uncoupling Protein type I* (*UCP-1*), *Peroxisome proliferator activated receptor* (*PPAR)γ 2* and *Apolipoprotein E* (*APOE*) [89].

Recently, Dongiovanni et al. demonstrated that *Proprotein Convertase Subtilisin/Kexin Type 7* (*PCSK7)* rs236918 G>C variant impacts on circulating lipids and liver damage in a large cohort of NAFLD patients [90]. In this cohort, the variant did not impact on hepatic fat accumulation, but stratifying patients for the PNPLA3 I148M risk variant, the rs236918 variation seems to be also associated with more severe steatosis. In particular, in HepG2 cells, PCSK7 genetic deletion reduced lipogenesis, fat accumulation, inflammation and fibrogenesis, even after the exposure to FFAs challenge [90]. Huang et al. investigated in 730 obese adults whether two-year weight-loss diets modified the effect of *PCSK7* rs236918 genetic variant on changes in fasting insulin levels and IR in the randomized, controlled Preventing Overweight Using Novel Dietary Strategies (POUNDS) LOST clinical trial (NCT00072995) [91]. The minor C allele was found significantly correlated with a strong increase in fasting insulin levels and HOMA-IR in response to high-carbohydrate diet consumption, providing novel information in guiding dietary intervention in diabetic patients. Nonetheless, alterations in *PCSK9*, another member of the Proprotein Convertase Subtilisin/Kexin family, have been broadly associated with familial hypercholesterolemia [92], severe steatosis [93] and cardiovascular risk [94], as a consequence of its role in the modulation of low-density lipoprotein (LDL) uptake. PCSK9 expression is strongly influenced by nutritional status. Indeed, PCSK9 mRNA levels impressively decrease in mice after 24 h of fasting and in turn, its expression is restored via SREBP1c activation upon high carbohydrate refeeding or insulin stimulation [95].

Finally, the rs4841132 G>A variant, enhancing the expression of *Protein Phosphatase 1 Regulatory subunit 3B* (*PPP1R3B*), which is involved in glycogen synthesis, has been recently reported to reduce the risk of NAFLD, but at the same time, may favor liver disease by facilitating glycogen accumulation [96,97]. Conversely, in a rodent model, hepatic genetic deletion of *PPP1R3B* significantly reduced glycogen synthase protein abundance, glucose incorporation into hepatic glycogen, total hepatic glycogen content and fasting plasma glucose compared to wild-type littermates [98]. Thus, a low-carbohydrate dietary approach and an active lifestyle in patients carrying the rs4841132 variant may improve hepatic glycogen accumulation.

## 4. Nutriepigenomics: Is It Worth Looking into? 

Epigenetic modifiers in NAFLD can represent novel molecular predictors, which can determine not only the early risk assessment, but also the disease progression and prognosis, providing a new perspective on NAFLD management. Epigenetics is a hereditable but reversible phenomenon that affects chromatin ultrastructure and transcription without modifying DNA sequence in response to environmental cues including DNA methylation, histone modifications and miRNAs targeting mRNA [6,9].

The emerging knowledge of *‘nutriepigenomics,’* referred to as the interaction between nutrients and genome through epigenetic mechanisms, is increasingly grabbing attention in the field of human complex diseases as MetS, neurological disorders and cancer [99]. The hypothesis of the Developmental Origins of Adult Health and Disease underlined that the exposure in utero to environmental stressors, such as diet, had intergenerational effects, compromising adult phenotype [100]. 

Hence, food intake could affect epigenome remodeling throughout life and, interestingly, several dietary habits could be critical during gestational and post-natal periods, leading to stable epigenetic changes, which, in turn, could impact metabolic disease susceptibility [6,99]. Likewise, it has been reported in both animals and humans that risk factors, such as maternal obesity, could predispose descendants to metabolic disorders due to an imprinted metabolic signature induced on microbiota during pregnancy [99]. Low protein (LP) diet administered in the second half of gestation has been associated with aberrant renin-angiotensin system as a consequence of epigenetic reprogramming, inducing CpG islands hypomethylation and miRNAs deregulation in the offspring [101]. Fat-enriched diets consumed in pregnancy may favor chronic-low grade inflammatory state into the placenta and in many organs of the offspring, among which the liver, leading to hepatic metabolic changes and contributing to steatosis. For instance, maternal HFD alters littermates’ DNA methylation, influencing genes involved in NASH pathogenesis and hepatic fibrosis [102,103]. 

Caloric restriction and lifestyle are the only available interventions for NAFLD management. Since epigenetics are reversible processes, selective diets based on specific micro/macro-nutrients modulating the epigenetic pattern could be encouraging for novel non-invasive and cost-effective approaches to ameliorate NAFLD and prevent its progression. Therefore, this chapter will summarize the main notions about diet-epigenome interplay that may be involved in the pathogenesis of NAFLD, paying attention to the lesser-known aspects, including the role of gut microbiota and the emerging therapies. 

### 4.1. DNA Methylation: from NAFLD towards HCC 

Aberrant pattern in DNA methylation may arise during NAFLD development, affecting nuclear DNA or mitochondrial DNA (mtDNA), and differentially methylated genes may disentangle simple steatosis from NASH [104]. For instance, it has been observed that biopsy-proven NASH patients had higher methylation levels of *Mitochondrially encoded NADH dehydrogenase 6* (*MT-ND6*) than individuals with simple steatosis and they correlated with NAFLD activity score (NAS) [105]. The enzymes involved in DNA methylation reaction are members of the DNA methyltransferases (DNMTs) family, which transfer a methyl group from *S*-adenyl methionine (SAM) to the fifth carbon of a cytosine (5mC) preceding a guanine nucleotide or CpG clusters. In humans, hepatic DNMT levels are increased in subjects affected by NASH and are significantly associated with histological liver damage severity [105]. Generally, CpG islands are enriched in promoter regions of the genes, thus representing a crucial mechanism that regulates gene expression [106]. Hypermethylation of CpG islands is generally linked to gene silencing, while hypomethylation of promoters may activate transcription. 

Deficiency in several dietary compound donors of methyl group, such as betaine, choline, vitamin B12 and folate, are associated with alterations in DNA methylation and may favor progressive NAFLD. Conversely, it has been shown that nutritional interventions and methyl donors’ supplementation improved body weight, insulin sensitivity and fat accumulation in the liver. Cordero et al. investigated the effects of methyl donors added to a high-fat-sucrose (HFS) diet on key regulatory genes involved in NAFLD. They found that HFS diet enriched of choline, betaine, vitamin B12 and folic acid induced f*atty acid synthetase* (*FASN*) hypermethylation in the livers of rats, improving TG accumulation [107]. Choline-deficient diet (ChDD) induced a reduction in PPARα expression, and its lower levels were correlated to high methylation in *PPARα* promoter [108]. Betaine, a lipotropic agent, has shown beneficial effects to revert HFD-induced hepatic steatosis acting on global methylome and, in detail, supports hypomethylation of CpG clusters in the *microsomal triglyceride transfer protein* (*MTTP*) promoter, which is involved in VLDL assembly and secretion [109]. Rats fed a diet without vitamin B12 and folate predominantly developed micro-vesicular steatosis, increased TG levels and impaired mitochondrial fatty acids β-oxidation, and these changes were due to the hypomethylation of *Peroxisome proliferator-activated receptor gamma coactivator 1-alpha* (*PCG1-α*). 

Methyl-deficient and obesogenic diets were also associated with far-reaching epigenetic changes leading to advanced liver injury and cancer development. Either calcium or magnesium deficient diets during gestation and nursing influence hepatic methylation of the *11β-hydroxysteroid dehydrogenase* (*Hsd11b*) gene type 1 and 2 of the offspring, contributing to prenatal susceptibility to IR and metabolic complications [110,111]. Dudley et al. suggested that hypomethylation of hepatic cell cycle inhibitor *Cyclin Dependent Kinase Inhibitor 1A (Cdkn1a)* is one of the earliest epigenetic changes occurring in pups born from HFD-fed dams predisposing the offspring to long-term hepatic dysfunction [112]. Rats fed a diet containing low methionine and lacking both choline and folic acid highlighted an early activation of the DNA repair genes and pronounced loss of DNA methylation that promote the progression to liver carcinogenesis [113]. 

Zeybel et al. isolated genomic DNA from percutaneous needle biopsies of patients with mild or severe NAFLD and identified significant differences across several CpGs islands within fibrosis-related genes, among which *transforming growth factor beta 1 (TGFβ1), Type I Collagen α1 (Col1A1)* and *Platelet-derived growth factor (PDGFα)* [114]. Notably, Murphy et al. observed 69,247 differentially methylated CpG sites in patients with advanced *versus* mild NAFLD and found a correlation between methylome data with transcriptome ones. In particular, hypomethylated genes matched with higher mRNA levels in advanced NAFLD individuals compared to those with mild NAFLD. Among these genes, altered methylation of *Fibroblast growth factor receptor 2* (*FGFR2*) and *Caspase 1* (*CASP1*), which are involved in epithelial to mesenchymal transition, inflammation and fibrotic processes, seemed to play a crucial role in the progression to cirrhosis and HCC [104]. 

### 4.2. Histone Modifications in NAFLD: Dietary Control of Chromatin Reorganization

Post-translational modification of histones is an essential mechanism to determine chromatin architecture, in terms of DNA packaging and accessibility, and gene expression. As the other epigenetic processes, it occurs in response to environmental cues and includes dynamic changes of aminoacidic residues in the histone tails as: 1) acetylation/deacetylation of lysine; 2) phosphorylation of serine, threonine and tyrosine; 3) methylation of the side chains of lysine and arginine; 4) other mechanisms among which deimination, ADP ribosylation, ubiquitylation, sumoylation, proline isomerization and the addition of β-N-acetylglucosamine [115].

Several enzymes that participate in histone modifications are associated with the epigenetic control of metabolic pathways. For instance, JHDM2A^-/-^ mice, which are deficient of *jumonji C (JmjC)-domain-containing histone demethylase 2A* (*Jhdm2a*), develop hallmarks of MetS, such as obesity, hyperglycaemia, hypercholesterolemia, hyperinsulinemia [116]. Fetal intrauterine growth restriction (IUGR) occurring during pregnancy and lactation determined biochemical abnormalities in adult rat offspring. Indeed, LP-fed rat dams led to high levels of cholesterol in the offspring and reduced the fetal expression of *Jhdm2a*, thus conditioning the hepatic lysine 9 acetylation of histone H3 (H3K9) in the *Cholesterol 7α-hydroxylase* (*Cyp7a1*) promoter, which catabolizes cholesterol to bile acids [117].

Sirtuin (Sirt) 1, involved in DNA repair, appetite control and in the crosstalk between adipose tissue and the liver, is considered a key epigenetic modifier in the pathophysiology of obesity and NAFLD. In response to caloric restriction, Sirt1 induces histone and non-histone deacetylation of its target genes, such as *PCG1-α* and *TP53* to adapt gene expression to metabolic activity, IR and inflammation in the adipose tissue. In the liver, Sirt1 supports gluconeogenesis via PCG1-α and Forkhead box O1 (FOXO1), promotes fatty acid oxidation through PPARα and inhibits DNL by SREBP1c degradation [118]. Bisphenol A (BPA) administered in Sprague Dawley rats during the pregnancy and breastfeeding period induced an increment of body weight and development of micro-vesicular steatosis in the male littermates further challenged with HFD. These metabolic alterations were coupled with sex-specific epigenetic modifications among which the reduced K4 dimethylation at histone H3 (H3Me2K4), which led to lower *carnitine palmitoyl transferase 1A* (*Cpt1a*) mRNA expression dysregulating mitochondrial β-oxidation. In addition, macroH2A1.1, a variant of histone H2A, whose expression is Sirt1-dependent, protects hepatocytes against lipid accumulation in mice [119,120]. Mice lacking *Sirt3* (SIRT3KO), the main mitochondrial protein deacetylase, accelerated obesity, IR and NASH development in response to chronic HFD exposure. According to these results, patients carrying the rs11246020 variant (V208I) in *SIRT3* gene increased susceptibility to develop MetS [121]. Furthermore, HFD *per se* suppressed Sirt3 expression in mice inducing global hyperacetylation of mitochondrial proteins [121]. 

Chung et al. found a novel histone acetyltransferase (HAT) inhibitor, the tannic acid (TA), a plant-derived hydrolysable tannin polyphenol and tested its efficacy in both in vitro and *in vivo*. TA administration showed anti-lipogenic effects, by blocking the hyperacetylation of lysine 9 and 36 at histone H3 (H3K9 and H3K36) in the promoter of DNL genes (SREBP1c, FASN and ATP citrate lyase (ACLY)), thus improving NAFLD [120,122]. Moreover, Tian et al. identified the histone deacetylase 8 (Hdac8) as a novel chromatin modifier in dietary models of NASH and HCC and in humans. Post-prandially activated Srebp1 lead to Hdac8 expression which interacts with the polycomb protein enhancer of zeste homolog 2 (Ezh2) to promote aberrant cell proliferation. Both in rodents and in patients with NAFLD-HCC, the HDAC8/EZH2 complex controls histone H4 acetylation and works as epigenetic silencing machinery on inhibitors of Wingless-related integration site (Wnt)/β-catenin signaling, thus favoring HCC development [123]. 

### 4.3. miRNAs-Diet Crosstalk in NAFLD: How Unhealthy Dietary Habits Act at Multiple Levels

miRNAs are short non-protein coding, single-strands RNAs of 19–22 nucleotides, that can target mRNAs through complementary base-pairing, thereby leading to the post-transcriptional repression of targeted protein-coding genes [124]. miRNAs can target multiple genes (multi-functionality) or multiple miRNAs can modulate a single gene (redundancy), suggesting that miRNAs have an extensive regulatory capacity and a profound impact on health and disease [124,125]. 

miRNAs may regulate a wide spectrum of biological processes and metabolic homeostasis, including lipid synthesis, fatty acids and glucose catabolism, inflammation, proliferation, apoptosis and necrosis, which have been known to be epigenetically deregulated in NAFLD, NASH and HCC [126]. Furthermore, it has been shown that circulating miRNAs may mirror the histological features and the molecular events occurring in NAFLD, thus, representing a turning point for non-invasive diagnosis and clinical monitoring of the disease progression [8,9,10,11,127,128]. Unhealthy diets affect adiposity and may accelerate NAFLD development in both animals and humans by altering miRNAs, which are susceptible to environmental challenge. We have previously reported in detail the miRNA signature associated with the spectrum of NAFLD, from simple steatosis towards cirrhosis and HCC [9]. Thus, in this chapter, we will summarize the main findings about miRNAs-diet alteration related to NAFLD onset and progression.

miR-122, the most abundant miRNA in human liver [9,129], has been proposed as a marker of NASH and disease severity and it has been demonstrated to be highly responsive to diet in in vitro and in vivo models as well as in humans. In genetically improved farmed tilapia (GIFT, *Oreochromis niloticus*) fed either a normal or high-lipid diet, inhibition of hepatic miR-122 led to upregulation of lipogenic genes, among which s*tearoyl-CoA desaturase gene* (*SCD*) at the 3′-UTR region, thereby affecting lipid metabolism and contributing to fat accumulation [130]. In contrast, Long et al. found that FFAs or HFD upregulated miR-122 in hepatocytes-derived HepG2 and HuH7 cell lines and in mice, respectively, revealing that miR-122 may promote lipogenesis by suppressing Sirt1. Indeed, miR-122 knockdown mitigated hepatic steatosis via Sirt1-induced liver kinase B1/AMP-activated protein kinase (LKB1/AMPK) pathway [131]. 

HepG2 cell line exposed to palmitic acid (PA) upregulates miR-181b, which acts as a repressor of SIRT1 mRNA at 3′-UTR. In presence of a mixture of fatty acids, human normal L02 hepatocytes increased miR-34a levels and, in parallel, downregulated its target genes [132]. Similarly, steatogenic diets as HFD reduced Sirt1 and PPARα expression and overexpressed both miR-181b and miR34a in mice livers, thus supporting the crucial role of these miRNAs to promote steatosis [132,133]. It has also been reported that miR-34a inhibits β-Klotho, a co-receptor of the fibroblast growth factor 19 (FGF19), implicated in glucose metabolism during post-prandial response in physiological status. The reduction of β-Klotho plasma levels has been recently associated with lobular inflammation, ballooning and fibrosis in NAFLD pediatric patients [134]. Therefore, the inhibition of miR-34a restores β-Klotho/FGF19 signaling and improves obesity in *ob/ob* mice [9,126,132]. It has been showed that miR-155 is highly expressed in hepatocytes and Kupffer cells (KCs) isolated from methionine-choline deficient (MCD)-fed mice [135,136], playing an important role in early hepatic lipid storage by targeting *LXRα* and favoring a pro-inflammatory response [135,136]. miR-21 is one of the most up-regulated miRNAs in serum and hepatic tissues of individuals with fibrosing-NASH and HCC [137,138]. Furthermore, it has been shown that lipogenic folate and choline-deficient diet induced NASH-specific changes of miRNAs, including miR-29c, miR-34a, miR-122, miR-134a, miR-155, miR-181a, miR-192, miR-200b, miR-409-3p, miR-410 and miR-495 in the livers of rodents [139,140]. 

We have recently explored the mechanisms through which genetics, epigenetics and environmental factors interact to promote advanced NAFLD in mice haplo-insufficient for the insulin receptor (InsR+/-) [8]. To this end, we performed whole transcriptome analysis in HSCs isolated from both wild-type and InsR+/− mice and found 36 differentially expressed miRNAs, which were implicated in metabolic, fibrotic and carcinogenic processes. Next, InsR+/- mice were fed an MCD diet to induce NASH, and we assessed the effect of diet-genotype interaction on hepatic miRNA expressions. We found that miR-101-3p downregulation significantly associated with the whole spectrum of liver damage and its reduction was not only InsR-dependent but even more exacerbated in the presence of MCD diet. Likewise, we confirmed these findings in both primary InsR+/- hepatocytes and HSCs isolated from mice and treated with fatty acids. Finally, we transfected HepG2 and LX-2 cells with miR-101-3p mimic and demonstrated that miR-101-3p overexpression dampened proliferation and migration, supporting the involvement of miR-101-3p in the epithelial mesenchymal transition to promote liver cancer [8]. 

## 5. Role of Gut Microbiota in NAFLD Pathogenesis and Possible Dietary-Based Strategies

The human gastrointestinal lumen is the largest reservoir of microorganisms in the body, representing the physiological habitat for more than 100 trillion microorganisms (bacteria, archaea, fungi, yeast and viruses) [141]. Among them, 85% of total bacteria are commensal microbes that live in synergy with the host, providing biological and metabolic functions. The majority of bacteria belongs to the phyla *Firmicutes* (Gram positive) and to *Bacterioidetes* (Gram negative), mainly involved in the short-chain fatty acids (SCFAs), i.e., acetate, butyrate and propionate and hydrogen production, respectively [10,11]. Although the crucial role of intestinal flora remains under definition, growing evidence demonstrates that microbial species are directly implicated in the processing and digestion of complex and indigestible polysaccharides to SCFAs, guaranteeing the energy supply to the host and the intestinal barrier preservation. Indeed, in physiological conditions, gut microbiota intervenes in the formation of these end-products from plant polysaccharides catabolism, which are then absorbed and delivered to the liver, where they are assembled in more structured lipids [10,11].

All abnormalities in intestinal flora taxonomic composition and/or function are usually referred to as ‘dysbiosis,’ a condition that has been largely explored in rodents and in NAFLD patients [10,11]. The dietary habits along with the caloric intake may strikingly contribute to the inter-individual variability of the intestinal bacterial strains. Indeed, a diet composition unbalanced in animal fat and sugars may more strongly increase the personal susceptibility to pathogenic bacteria over-growth, exerting a detrimental effect on the immunological tolerance of mucosal cells, as shown in a large number of preclinical [142,143] and clinical studies [144,145]. In particular, Western diet and HFD have been related to the increased amount of pro-inflammatory bacterial species, altering gut barrier integrity, intestinal pH and lipopolysaccharide (LPS) transition into the blood flow (endotoxemia) [146]. Indeed, the intestinal barrier is constituted by tight and adherent junctions and desmosomes, which hold together the epithelial cells and regulate the bidirectional flux between the gut and the liver. Specifically, intestinal barrier protects the host from pathogen invasions and impedes microbial systemic translocation [10]. Therefore, the erosion of the protective mucus layer, the reduction of antimicrobial mediators and microbial dysbiosis have been correlated with viable pathogenic bacteria, Gram-negative microbial products and pro-inflammatory luminal metabolites translocation into the blood circulation, contributing to liver damage [147,148]. The increased concentration of circulating LPS leads to the activation of Toll-like receptor 4 (TLR4)/nuclear factor kappa-light-chain-enhancer of activated B cells (NF-κB) signaling, which drives, in turn, pro-inflammatory cytokines release, reactive oxygen species (ROS) production and oxidative stress. This event cascade may trigger the activation of resident macrophages, the KCs and HSCs, further corroborating inflammation and fibrosis into the liver [135,149]. Moreover, several endogenous molecules, such as as ethanol, ammonia and acetaldehyde, whose circulating increased levels result from dysbiotic microbiota (i.e., *Escherichia coli* abundance), are able to stimulate hepatic KCs to produce pro-inflammatory cytokines with similar mechanisms occurring in alcohol-induced liver damage [10,150].

An impairment in gut barrier function and a decrease in SCFA-producing agents have also been observed after a chronic fructose consumption, which foster macrophage activation in the liver through TLRs and Myeloid differentiation factor 88 (Myd88)-dependent pro-inflammatory pathways in mice [151]. Similarly, acute and chronic high fructose administration exacerbated endotoxemia in pediatric NAFLD patients and correlated with liver inflammation [152].

Dietary modifications can rapidly normalize intestinal microbiota, thus representing a simple and effective approach to restore eubiosis. Indeed, the diet is enabled to profoundly reshape the microbiota composition within a few hours. People consuming a Western diet and subjects with high-fiber dietary habits display a tremendous difference in microflora taxonomic composition, as shown in an elegant study in which American volunteers were randomized to receive an animal-based diet (meats, eggs and cheese) or a plant-based diet (cereals, legumes, fruits and vegetables). In individuals under animal-based regimen, an increase in bile-tolerant species such as *Alistipes, Bilophila* and *Bacteroides* and a reduction of *Roseburia, Eubacterium rectale* and *Ruminococcus bromii,* which metabolizes dietary plant polysaccharides, was observed [153,154]. In this context, a diet supplemented in fibers may favor enormous benefits for health as a consequence of their intestinal fermentation into SCFAs, mediated by colonic bacteria. Indeed, fecal samples of vegan and vegetarian individuals showed a significant decrease in *Bidifobacterium* and *Bacteroides* species, as well as Africans fed a high-carbohydrate vegetarian diet compared to English subjects assuming a mixed Western diet [155,156]. Nonetheless, a diet enriched in fibers and carbohydrates lowered fecal pH, mainly due to the products of gut fermentative metabolism and the hampered growth of pathogens along with *Escherichia coli* and *Enterobacteriacee* [157,158].

Finally, natural extracts, such as polyphenols provided by coffee, green tea and chocolate, have been demonstrated to induce beneficial effects by directly interacting with gut microbial communities. In C57Bl/6 mice fed HFD, grape polyphenols administration improved insulin sensitivity, attenuated inflammation and ameliorated intestinal barrier integrity. Overall, diets enriched in phenols have been associated with improved MetS features and immune tolerance, and with the restoration of intestinal barrier function, by promoting eubiosis. 

### Changes in Gut Microbiota Species Alters the Hepatic Epigenetic Landscape: A Tipping Point 

It is a common knowledge that nutrition strongly influences microbiome composition, but it is less known that there is a long-term and persistent impact of intestinal bacteria changes on hepatic epigenome, a mechanism termed the “*priming effect*.” Recently, Kim et al. reported that mice feeding either HFD or a high-fructose diet (HFrD) increased serum cholesterol, developed hepatic steatosis and showed an enrichment in the *Odoribacter*, which produces butyrate. When both HFD and HFrD groups were exposed to normal chow (NC), mice rescued only certain pathological features, such as lower body weight, improved glucose tolerance and reduced fat accumulation in the liver. Interestingly, *Odoribacter* remained enriched after NC and high levels of butyrate were correlated to persistent changes in liver DNA methylation, probably due to butyrate ability to act as CpG islands modifier, thus, suggesting a possible link between microbial by-products and nutriepigenomics [102,159]. 

**Figure 1:**
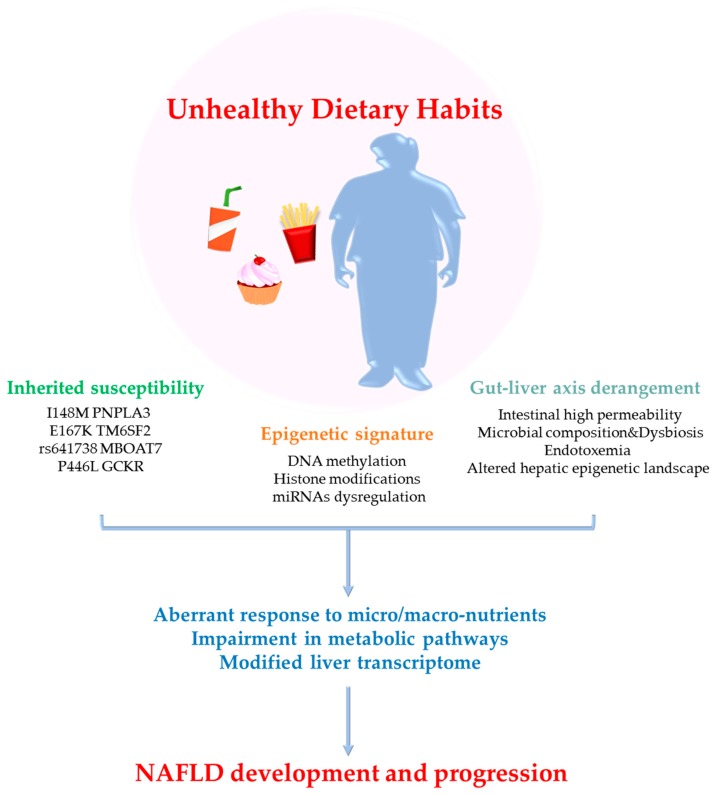
Nutrition, genetic and epigenetic crosstalk in nonalcoholic fatty liver disease (NAFLD).

Kimberly et al. had demonstrated that the composition of gut microbiota altered global histone acetylation and methylation in host tissues in a diet-dependent manner. Mice fed a high-fat/high-sucrose (HF/HS) diet, which contains low levels of fermentable complex polysaccharides, showed loss of cecal SCFAs production (acetate, propionate and butyrate) compared to NC mice [160,161]. The reduction of SCFAs correlated to profound post-translational modification of hepatic histones, such as lower methylation of H3 histones in specific aminoacidic position (H3K27me1 and H3K36me2) [160,161]. The impact of unhealthy dietary habits on NAFLD and their interaction with inherited and acquired risk factors is schematically represented in Figure 1.

Bioactive compounds contained in food actively contribute to physical welfare and could provide benefits as preventive and healing molecules for metabolic disorders, such as obesity, T2D and NAFLD. Conversely, unhealthy dietary habits could affect metabolic homeostasis acting at multiple levels. Chronic exposure to high fat/carbohydrate diets could increase intestinal barrier permeability, favor dysbiosis and raise the amount of circulating bacterial endotoxins, thus, unbalancing gut-liver axis bidirectional flux of metabolites. Interestingly, several micro/macro-nutrients may specifically enrich certain bacterial species, such as *Odoribacter*, which are involved in changes of the hepatic epigenetic landscape. In the liver, the presence of genetic variants predisposing to NAFLD development and progression (I148M PNPLA3, E167K TM6SF2, the rs641738 in *MBOAT7* gene, the P446L GCKR and others) may alter the effectiveness of beneficial nutrients or accelerate the effects of unhealthy by-products derived from junk food. Similarly, epigenetic modifiers (DNA methylation, histone modifications and miRNAs regulation) could be highly nutrient-sensing, as they respond to environmental cues and could establish long-lasting effects from the gestational and post-natal period onward. The high complex network made by the combination of the environment, genes and epigenetics may cause aberrant downstream responses to nutrients, determining NAFLD onset and progression. 

## 6. Precision and Accuracy: The Dartboard of Nutrition 

If on the one hand junk food and a sedentary lifestyle cause metabolic dysfunction, on the other hand, the study of nutri-genetics/epigenetics enabled us to identify either different genetic polymorphisms, which may modulate the effectiveness of nutrients, and epigenetic markers that may be potential therapeutic targets of specific dietary interventions. Livingstone et al. investigated the effects of personalized nutrition in a European randomized controlled trial (Food4Me) (NCT01530139). Among the 1607 participants, 448 were overweight and the internet-based personalized diet favored the reduction of body weight and influenced dietary changes by enhancing the consumption of vegetarian diet [162]. 

Bioactive substances, such as polyphenols, flavonoids, fish-derived oils and, in general, compounds enriched in the MedDiet, predominantly consisting of fruits and vegetables, have shown systemic benefits as preventive and curative molecules for metabolic diseases, cardiovascular risk and cancer [163,164,165,166]. Regular intake of MedDiet with coenzyme Q10 (Med + CoQ diet) has been associated with a lower expression of genes involved in inflammation, ER/oxidative stress and DNA damage in the peripheral blood mononuclear cells (PMBCs) of 63 volunteers [166]. Yubero-Serrano et al. enrolled 20 individuals who were randomized to receive Med + CoQ for 4 weeks. They found that Med + CoQ protects against ROS overproduction through mitigating the expression of oxidative, inflammatory and fibrotic markers, such as superoxide dismutase (SOD) 1/2 and matrix metalloproteinase (MMP) 9 [165]. Interestingly, either bariatric surgery or energetic restriction through MedDiet, used as obesity treatment, may also epigenetically modulate the expression of some of these markers influencing global DNA methylation and hydroxymethylation [164,167]. A high-protein diet improved hepatic steatosis in a mouse model of NAFLD independently of high fat and carbohydrate intake by increasing the lipid and branched-chain amino acid (BCAA) catabolism, enhancing mitochondrial oxidative ability and reducing fatty acid desaturation, ER stress and unfolded protein response [168]. α-lipoic acid (ALA), a potent endogenous antioxidant and anti-inflammatory factor, has been shown to improve insulin sensitivity and fatty liver in 50 NAFLD subjects enrolled in a double-blinded controlled trial [169]. In addition, ALA administration blocks hepatic TGFβ mediators, stimulates MMP13 activity and actively participates to mitochondrial turnover inhibiting HDCA6, thus preventing hepatic damage and fibrosis [170,171]. The supplementation of n-3 fatty acids has been observed to accelerate TG clearance by augmenting the transcription rate of the PPARα-induced lipoprotein lipase activity (LPL) and maternal n-3 administration may influence the predisposition of IR and T2D of the offspring [172,173]. However, the occurrence of the PPARα L162V polymorphism hampers n-3 beneficial effects both in HepG2 cells and humans [172]. 

Apple polyphenols and red wine extracts as resveratrol and derivates have been shown to epigenetically prevent diet-induced obesity and ameliorate liver injury and cardiac dysfunction [174,175,176,177]. For instance, curcumin acts as a free radical scavenger and hampers lipid peroxidation and oxidative DNA damage. In a randomized double-blind placebo-controlled trial, the short-term curcumin administration in NAFLD patients improved hepatic fat content and metabolic profile (trial registration IRCT20100524004010N24) [178]. In addition, it has been demonstrated that curcumin works as an HAT inhibitor specifically targeting p300/CREB-binding proteins [176] and reduces the expression of DMT1 and pro-fibrotic markers (α smooth muscle actin (αSMA), Col1α1) in rodents affected by carbon tetrachloride (CCL4)-induced liver fibrosis, thus exerting hepatoprotective effects on fibrogenic processes [177]. Green tea, rich in polyphenols and catechins, is a natural hypolipidemic, antioxidant and thermogenic agent whose beneficial effects on hepatic steatosis and liver damage have been widely studied in both genetically and dietary induced experimental models of NAFLD/NASH [179,180,181,182,183,184,185]. One of the mechanisms by which green tea protects against NAFLD is linked to miR-34a and miR-194 downregulation accompanied by an increase in their mRNA targets, such as Sirt1 and Pparα [186]. 

### Nutri-Genetics/Epigenetics: A Smart Use of Nutrients in Ongoing Trials

According to the current knowledge that several nutrients could provide healing effects or rescue some of the pathological features of the MetS, some clinical trials have been conducted to explore gene-diet interactions as a plausible strategy to manage NAFLD/NASH. An observational study is recruiting participants affected by obesity, diabetes, NAFLD and other metabolic diseases to evaluate the effects of folates and the epigenetic mechanisms on severe obesity-related complications pre- and post-bariatric surgical interventions (NCT02663388; OBESEPI). In a randomized-controlled trial (NCT03183193) overweight NAFLD patients will receive FLiO diet, based on the distribution of macronutrients as follows: 30%–35% lipid (extra virgin olive oil and fatty acids Ω3), 25% vegetable proteins and 40%–45% fibers. As goals of this project, anthropometric and clinical outcomes will be evaluated as well as gut microbiota composition and epigenetic changes in terms of DNA methylation and miRNAs signature will be assessed to identify non-invasive biomarkers for early diagnosis in obese individuals. Many bioactive substances in fruits, vegetables and plants have antioxidant, anti-inflammatory, antimicrobial and lipid-lowering properties. Mastiha, a Greek liqueur made with mastic resin, is under investigation in a multicenter, randomized, double-blind, placebo-controlled trial (NCT03135873), named MAST4HEALTH. This study aims to assess the effect of Mastiha supplementation on the clinical course of NAFLD/NASH patients and correlating genetic/epigenetic markers with metabolomic and intestinal microbiota profiles. Interestingly, obese adults with dyslipidemia and obese children with IR will be recruited in the NutriGen project (NCT02837367). In this randomized triple-blind study, a nutrigenomic model will be exploited to determine the effectiveness of treatments with specific dietary foods (supplements containing methyl-donors), based on the genetic risk predisposition (genetic signature) of obese individuals. A schematic description of the main clinical trials described in the present review is represented in Table 1.

## 7. Concluding Remarks

The Western human diet has evolutionally changed, and nowadays, it is markedly enriched in saturated and trans-fat, omega-6 fatty acids, carbohydrates and high-energy nutrients against fruits, vegetables, proteins and omega-3 fatty acids [14]. Nutritional genomics address to study the gene-environment interactions and the detrimental effect of the changes in our dietary landscape. It may represent a promising tool to revolutionize both clinical and public health nutrition practice and may favor the establishment of genome-informed nutrient and food-based dietary guidelines for disease prevention and a healthy lifestyle, individualized medical nutrition therapy for NAFLD management and better targeted health nutrition interventions, including micronutrient supplementation, maximizing the benefits and in turn minimizing the adverse outcomes within genetically diverse human populations [13]. In this context, the study of nutri-epigenetics is becoming increasingly attractive, as it would allow to identify novel appealing bioactive compounds, which may contribute specifically to modulate the hepatic epigenetic signature from maternal and lactation period onward.

To date, no therapeutic strategy is approved for the treatment of NAFLD, and lifestyle modifications, physical exercise and weight loss remain the cornerstone of approaches to patients with NAFLD. Indeed, personalized nutritional recommendations for NAFLD patients remain largely unexplored and the deep understanding of the mechanisms behind gene–environment interactions should be a priority for future researches. Considering nutrigenomics as an option will guarantee us the clef to compose the harmonic combination of nutrients suitable for our genome, orchestrating the perfect symphony of the health.

## Figures and Tables

**Table 1:** Clinical Trials Addressing Nutrigenomic Interventions in NAFLD Patients.

Clinical Trial Start-End Date	Status	Study Type	Interventions	Conditions	Objectives	Locations
NCT00760513 11/09-11/18	Completed (*n* = 103)	Interventional Randomized Phase IV	Omega-3-acid (OMACOR) 4 gr/d oral capsule for 18 months versus placebo	Biopsy-verified NASH or Ultrasound-verified NAFLD	Identify the effects of n-3 fatty acids on fibrosis and cardiovascular risk biomarkers in NAFLD patients	Southampton General Hospital Southampton, Hants, United Kingdom
NCT00885313 03/09–03/11	Completed (*n* = 60)	Interventional Randomized Phase II	DHA 250 or 500mg/kg/d oral for 24 months vs. placebo	NAFLD and obesity in children	Evaluate the effects of DHA on Children With NAFLD	Bambino Gesù Hospital and Research Institute Rome, Italy
NCT00697580 05/059–06/07	Completed (*n* = 104)	Interventional Randomized	Behavioral: Nutrition changes and physical exercise	NAFLD and obesity in adolescents	Investigate the Intra-Abdominal Fat, insulin sensitivity and Risk of Disease in Adolescents	Veronica Atkins Lifestyle Intervention Laboratory Los Angeles, CA (USA)
NCT00072995 09/03–09/07	Completed (*n* = 811)	Interventional	Four Diets Differing in Macronutrient Composition and Low in Saturated Fat	Cardiovascular disease, NAFLD, obesity	Preventing Obesity Using Novel Dietary Strategies	Pennington Biomedical Research Center, Louisiana State University, Baton Rouge, LA (USA). Harvard University School of Public Health, Boston, MA (USA)
NCT01530139 02/12–02/16	Completed (*n* = 1607)	Interventional Randomized	Three personalized diets (Food4Me) versus control diet	Adult individuals	Personalization of dietary advices based on biochemical and/or genetic information	Universities of Newcastle; College Dublin; Maastricht; Oslo; Navarra; Harokopio; Reading; München; Newcastle Upon-Tyne and National Food and Nutrition Institute
NCT02663388 01/16–12/19	Recruiting (*n* = 20,000) *	Observational	Gastric bypass, sleeve, gastric banding	Cardiovascular disease, NAFLD, obesity, MetS, T2D	Evaluate DNA Methylation on severe obesity-related complications before and after bariatric surgery	University of Lorraine, CHU Nancy Vandoeuvre Les Nancy, France
NCT03183193 06/16–12/19	Recruiting (*n* = 120) *	Interventional Randomized	FLiO diet versus control diet	NAFLD and obesity	Nutritional/lifestyle interventions to identify non-invasive biomarkers to early diagnosis of NAFLD in future obese people	Centre for Nutrition Research, University of Navarra Pamplona, Navarra, Spain
NCT03135873 03/17–12/19	Recruiting (*n* = 52) *	Interventional Randomized (Phase I)	Mastiha vs. placebo	Obese NAFLD	Explore gene-diet interactions and correlate genetic/epigenetic markers with metabolome and gut microbiota	Harokopio University, Athens, Attica, Greece
NCT02837367 06/19–12/19	Recruiting (*n* = 600) *	Interventional Randomized	Administration of supplements containing methyl-donors	Obesity and dyslipidemia	Explore the genetic signature involved in the donation of methyl groups and unsaturated omega-6/3 fatty acid metabolism	Clinica II Pediatrie Bega Timisoara, Timis, Romania. Spitalul Judetean Timisoara; Centrul de Diabet Timisoara, Timis, Romania

* Estimated number of participants.

## Abbreviations

5mC	5-methylcytosine
αSMA	α smooth muscle actin
ACDRP	Amish Complex Disease Research Program
ACLY	ATP citrate lyase
ALA	α-lipoic acid
ALT	Alanine Aminotransferase
APO C3	Apolipoprotein C3
APOE	Apolipoprotein E
b3AR	Adrenergic receptor
BCAA	Branched-Chain Amino Acid
BPA	Bisphenol A
CASP1	Caspase 1
CBMN	Cytokinesis-block micronucleus assay
CCL4	Carbon Tetrachloride
Cdkn1a	Cyclin Dependent Kinase Inhibitor 1A
ChDD	Choline-deficient diet
Col1A1	Collagen Type I α1
CPT1A	Carnitine Palmitoyltransferase 1A
CRISPR/Cas9	Clustered regularly interspaced short palindromic repeats
Cyp7A1	Cholesterol 7-α hydroxylase 1
DHA	Docosahexaenoic acid
DNL	*De novo* lipogenesis
DNMTs	DNA methyltransferases
ELISA	Enzyme-linked immunosorbent assay
ER	Endothelial Reticulum
EPA	Eicosapentanoic acid
EZH2	Enhancer of Zeste Homolog 2
FASN	Fatty acid synthetase
FATP1	Fatty acid transporter 1
FA	Fatty Acid
FFA	Free Fatty Acid
FGF19	Fibroblast Growth Factor 19
FGFR2	Fibroblast growth factor receptor 2
FOXO1	Forkhead box O1
GCKR	Glucokinase Regulatory Protein
GIFT	Genetically Improved Farmed Tilapia
GSTM1	Glutathione S-transferase Mu 1
GSTT1	Glutathione S-transferase theta 1
GWAS	Genome-wide association study
H3K9	Lysine 9 acetylation of histone H3
H3K36	Lysine 36 acetylation of histone H3
H3Me2K4	Lysine 4 dimethylation at histone H3
HAT	Histone acetyltransferase
HCC	Hepatocellular Carcinoma
HDAC8	Histone Deacetylase 8
HFD	High Fat Diet
HFrD	high-fructose diet
HF/HS	High-Fat/High-Sucrose
HFS	High Fat Sucrose
HSCs	Hepatic Stellate Cells
HSD11B	11β-hydroxysteroid dehydrogenase
iPOP	Integrated personal omic profile
IR	Insulin Resistance
IUGR	Intrauterine Grow Restriction
JHDM2A	Jumonji C (JmjC)-Domain-containing Histone Demethylase 2A
LDL	Low-density lipoprotein
LKB1/AMPK	Liver Kinase B1/AMP-activated Protein Kinase
LP	Low protein
LPIAT1	Lyso-phosphatidylinositol acyl-transferase1
LPL	Lipoprotein lipase activity
LPS	Lipopolysaccharides
LXRα	Liver X receptor alpha
Lyso-PI	Lyso-phosphatidylinositol
MAM	Mitochondria-associated membrane
MBOAT7	Membrane Bound O-acyltransferase Domain-containing 7
MCD	Methionine-Choline Deficient diet
MedDiet	Mediterranean diet
MetS	Metabolic Syndrome
miRNAs	microRNAs
MMP	Matrix Metalloproteinase
mtDNA	Mitochondrial DNA
MTHFR	Methylenetetrahydrofolate reductase
MT-ND6	Mitochondrially encoded NADH dehydrogenase 6
MTTP	Microsomal triglyceride transfer protein
MUFA	Monounsaturated fatty acid
Myd88	Myeloid differentiation factor 88
NAFLD	Nonalcoholic Fatty Liver Disease
NAS	NAFLD activity score
NASH	Nonalcoholic Steatohepatitis
NC	Normal Chow
NF-κB	Nuclear Factor Kappa-Light-Chain-Enhancer of Activated B Cells
NIOR	Nutrient-induced insulin output ratio
NMR	Nuclear Magnetic Resonance
PA	Palmitic acid
PCG1-α	Peroxisome proliferator-activated receptor gamma coactivator 1-alpha
PCSK7	Proprotein Convertase Subtilisin/Kexin Type 7
PCSK9	Proprotein Convertase Subtilisin/Kexin Type 9
PDGFα	Platelet-derived growth factor alpha
PMBCs	Peripheral blood mononuclear cells
PNPLA2	Patatin-like Phospholipase Domain Protein 2
PNPLA3	Patatin-like Phospholipase Domain Protein 3
PPARα	Peroxisome proliferator-activated receptor α
PPARγ 2	Peroxisome proliferator-activated receptor γ2
PPP1R3B	Protein Phosphatase 1 Regulatory subunit 3B
PUFA	Polyunsaturated fatty acids
RNAseq	RNA sequencing
ROS	Reactive Oxygen Species
SAM	S-adenyl methionine
SCD	Stearoyl-CoA Desaturase
SCFAs	Short Chain Fatty Acids
SFA	Saturated fatty acid
Sirt	Sirtuin
SNPs	Single nucleotide polymorphisms
SOD	Superoxide Dismutase
SREBP1c	Sterol Regulatory Element Binding Protein 1c
SULT1A1	Sulfotransferase 1A1
T2D	Type 2 Diabetes Mellitus
TA	Tannic acid
TG	Triglycerides
TGFβ1	Transforming growth factor β1
TLR4	Toll-like receptor 4
TM6SF2	Transmembrane 6 Superfamily Member 2
TMC4/MBOAT7	Transmembrane Channel-like 4/Membrane Bound O-acyltransferase Domain-containing
TNFα	Tumor Necrosis Factor alpha
UCP-1	Uncoupling Protein type I
VLDL	Very-low density lipoprotein
WNT	Wingless-related integration site
	
